# Camera-traps are a cost-effective method for surveying terrestrial squamates: A comparison with artificial refuges and pitfall traps

**DOI:** 10.1371/journal.pone.0226913

**Published:** 2020-01-16

**Authors:** Dustin J. Welbourne, Andrew W. Claridge, David J. Paull, Frederick Ford

**Affiliations:** 1 Department of Wildlife Ecology and Conservation, University of Florida, Gainesville, Florida, United States of America; 2 School of Science, University of New South Wales, Canberra, Australian Capital Territory, Australia; 3 NSW Department of Primary Industries, Vertebrate Pest Research Unit, Queanbeyan, New South Wales, Australia; 4 Office of Environment and Heritage, National Parks and Wildlife Service, Nature Conservation Section, Queanbeyan, New South Wales, Australia; 5 Estate and Infrastructure Group, Department of Defence, Canberra, Australian Capital Territory, Australia; University of Southern Queensland, AUSTRALIA

## Abstract

**Introduction:**

Fundamental data on the distributions, diversity, and threat status of terrestrial snakes and lizards (hereafter squamates) is limited. This is due to the cryptic nature of species in this faunal group, and to limitations in the effectiveness of the survey methods used to detect these species. Camera-traps are a useful tool for detecting numerous vertebrate species, yet their use for detecting squamates has been limited. Here, we apply recent methodological advancements in camera-trapping and assessed the utility of camera-traps for inventorying a squamate assemblage by comparing camera-trapping survey results with two widely used labour-intensive methods: artificial refuges and pitfall traps.

**Methods:**

We conducted a 74-day survey using camera-traps and, concurrently, four by four-day surveys using labour-intensive methods. Given the duration and three detection methods, we compared seven variants of survey protocol, including using each method alone or all methods simultaneously. We compared both the effectiveness and cost-effectiveness of each survey protocol by estimating the number of species detected at the transect level, and by calculating the costs of conducting those surveys.

**Results:**

We found the camera-trapping survey was most cost-effective, costing 687 AUD (CI 534–912) per squamate species detected, compared with the 2975 AUD (CI 2103–4486) per squamate species detected with the labour-intensive methods. Using all methods together was less cost-effective than using camera-traps alone. Additionally, there was a 99% probability that camera-traps would detect more species per transect than the labour-intensive methods examined.

**Discussion & conclusion:**

By focusing the analysis at the level of the survey, rather than the level of the device, camera-traps are both a more effective and cost-effective technique for surveying terrestrial squamates. Where circumstances are appropriate, those wildlife researchers and managers currently using camera-traps for non-squamate surveys, can adopt the methods presented to incorporate squamate surveys with little upfront cost. Additionally, researchers currently using traditional techniques can be confident that switching to camera-traps will likely yield improved results. Still, camera-traps are not a panacea and careful consideration into the benefits and usefulness of these techniques in individual circumstances is required.

## Introduction

Snakes and lizards (hereafter squamates) are one of the most speciose groups of terrestrial vertebrates [1]. Yet, compared with mammals or birds, little is understood about the roles squamate play in ecological systems or their threat status. For example, the IUCN [2] has been unable to provide an estimate of the proportion of reptiles that are threatened globally due to data deficiency. This is problematic given the comparatively high sensitivity of reptiles to threats such as habitat loss [3]. While there are numerous reasons for the paucity of fundamental data on squamates, the general difficulty of detecting them efficiently is foremost. This difficulty stems from the cryptic nature of most squamates and the limitations of existing survey methods used to detect them. Survey methods that are cost-effective and produce reliable data are fundamental to detecting and managing all wildlife populations. Given this deficiency, there is a clear need to develop cost-effective techniques for detecting squamates.

Camera-traps have become an increasingly useful tool for detecting various vertebrates and answering a range of research questions [4]. Although camera-traps are used mostly to detect mammals their utility for detecting reptiles generally and squamates specifically has recently grown [5]. For reptiles, camera-traps have been principally used to monitor individual squamate species (*e*.*g*. *Varanus komodoensis*; [6]), with limited use for surveying squamate assemblages. Welbourne [7] demonstrated how passive-infrared (PIR) triggered camera-traps can reliably detect a range of squamate species, including small specimens, and demonstrated the technique was as effective as artificial refuges to detect squamates in a temperate environment [8]. Comparisons in semiarid environments similarly demonstrated that PIR triggered camera-traps were as effective as pitfall traps for inventorying diurnal squamates, although limited for nocturnal species [9]. Adams *et al*. [10] used time-lapse triggered camera-traps in long-leaf pine savannah and concluded camera-traps were a cost-effective alternative to box-traps for detecting terrestrial squamates. These results are encouraging and suggest further development of camera-trapping methods, and comparisons with existing standard survey techniques for detecting squamates, are warranted. By further improving camera-trapping methods for squamates, they offer a real substitutable survey method for monitoring squamates.

As squamates are morphologically diverse and exploit various niches, numerous survey methods have been variously devised for detecting them [11]. Using multiple, complementary detection methods is typically most effective [12] and most cost-effective [13] for determining squamate diversity. Thus, comparing the cost effectiveness of camera-traps to well-established complementary methods is a logical next step. Pitfall traps and artificial refuges are two methods commonly employed to detect terrestrial squamates. Artificial refuges “effectively sample snakes and lizards that are small relative to the size of the cover objects, [in addition to] species that seek cover beneath surface objects in the landscape” [14]; and, pitfall traps with drift-fences are “one technique…that detects all or most species in the area with minimal sampling bias” [15]. These methods are also complementary. Lettink and Cree [16] found geckos were detected more effectively with artificial refuges whereas skinks were more effectively detected with pitfall traps.

To examine the cost-effectiveness of camera-traps for detecting a squamate assemblage, we compared outcomes and costs of conducting squamate surveys with camera-traps and two often used complementary labour-intensive methods, artificial refuges and pitfall traps. We determined the probability that one survey protocol would detect more squamate species, at the transect level, than another to evaluate the effectiveness of each technique. By determining survey costs, we evaluated cost-effectiveness of the different approaches. Although surveys were conducted in a temperate heathland on the south-east coast of Australia, the results are nevertheless broadly applicable to similarly structured environments.

## Materials and methods

### Survey design

We conducted the study on Beecroft Weapons Range (BWR), ~135 km south of Sydney, Australia. Beecroft Weapons Range has a temperate climate and is dominated by coastal heath from less than a metre to above two metres in height. The camera-trap and labour-intensive methods surveys were conducted concurrently between 23 November 2014 and 4 February 2015. Forty transects (~2 x ~100 m) that had vegetation cleared to understory level had been established across BWR perpendicular to vehicle tracks, starting ~2 m from track edges. However, only 10 transects could be surveyed due to site access and equipment constraints.

### Camera-trapping protocol

**Fig 1:**
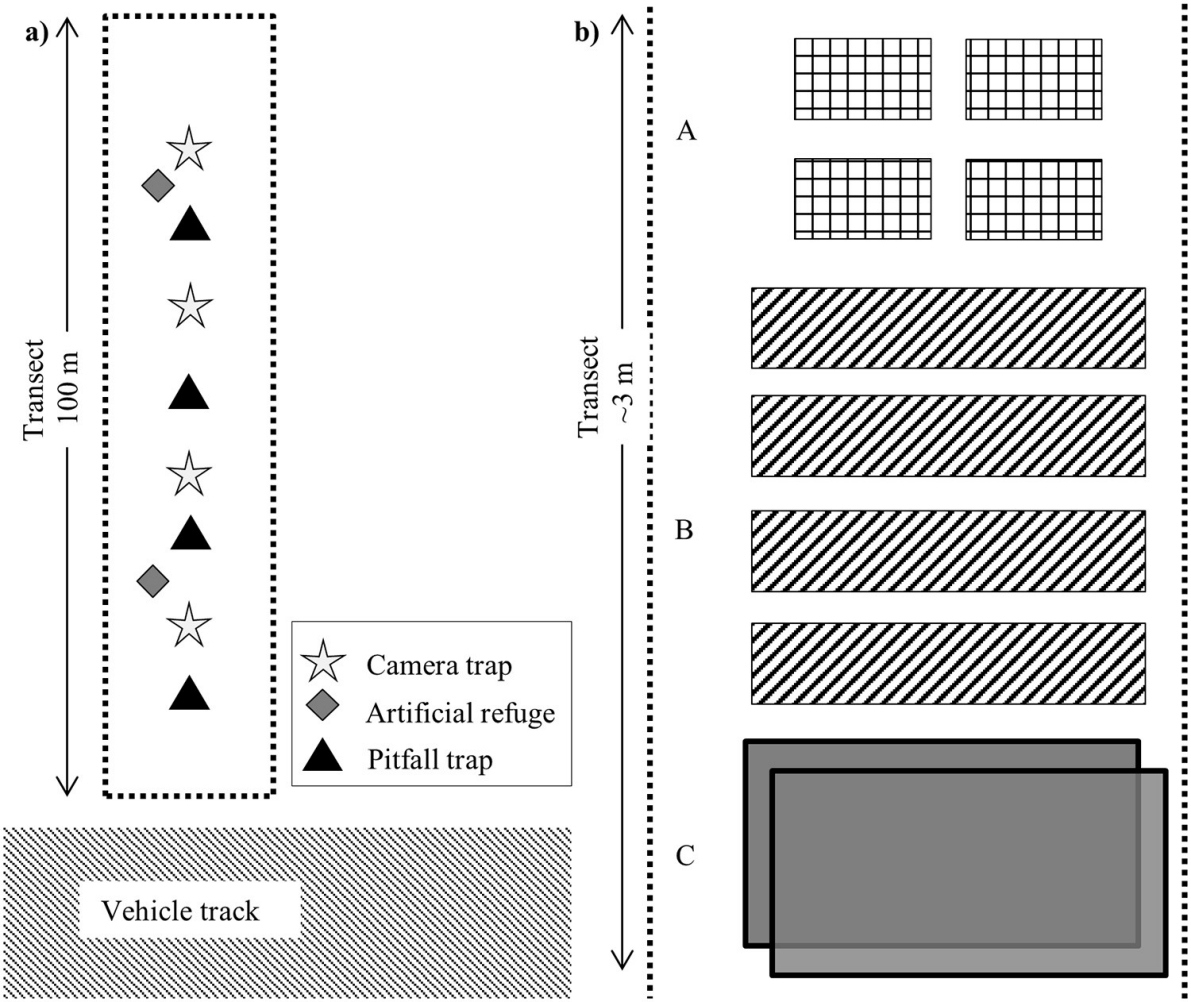
Layout of camera trap and labour-intensive survey devices on transects. Image a) is the spatial configuration of devices along the transect, and b) the configuration of artificial refuge arrays over ~3 m of transect. Artificial refuge arrays consisted of: A, concrete roof tiles; B, railway sleepers; and C, a pair of corrugated steel sheets stacked one on top of the other.

**Fig 2:**
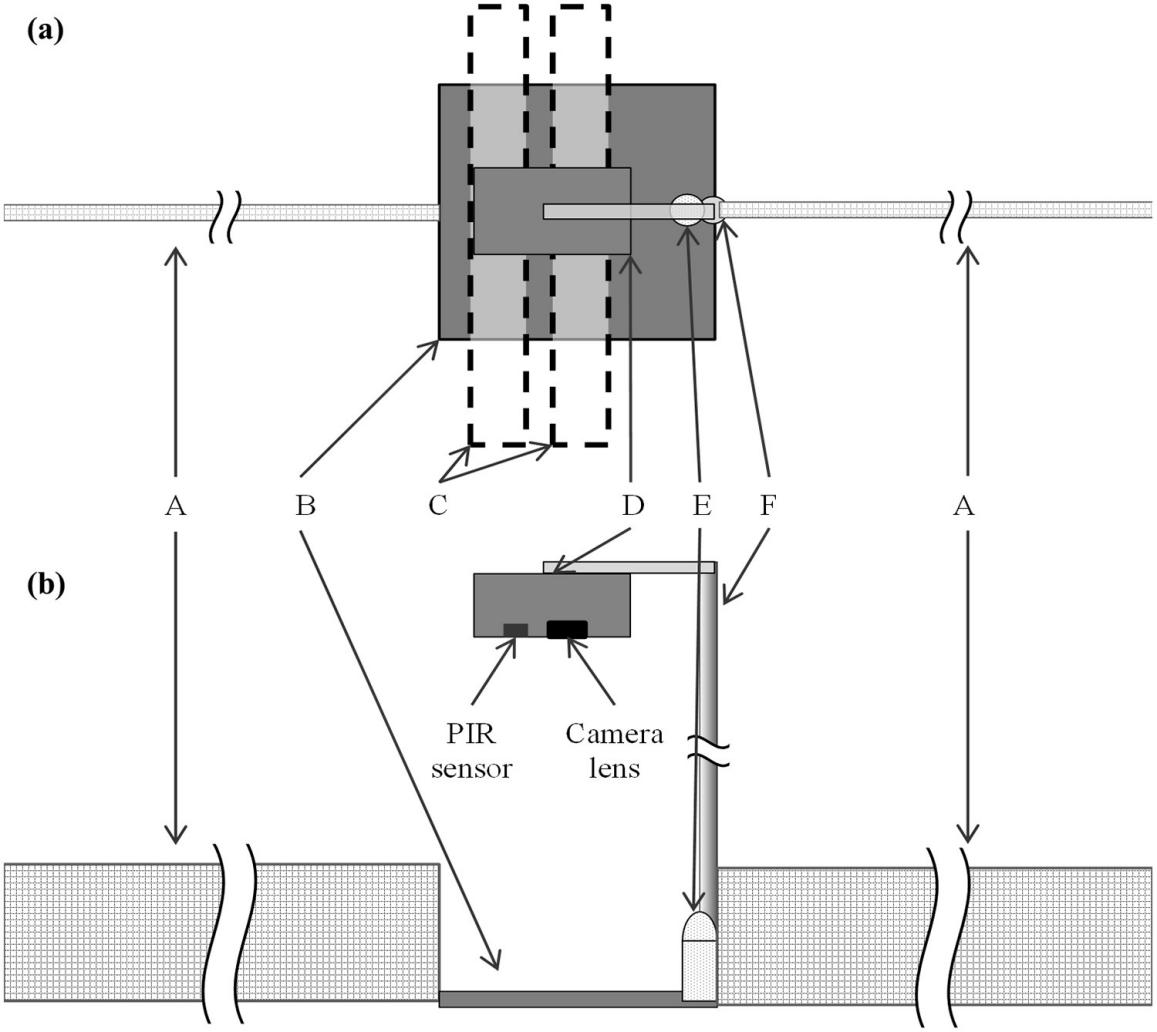
Camera-trap station setup. The a) plan view and b) profile view of the camera trap station setup. Components are: A, drift fence (~30 x ~300 cm); B, cork floor tile (30 x 30 x 0.6 cm); C, passive infrared (PIR) detection bands to demonstrate orientation; D, Reconyx HC600 camera trap; E, PVC bait-holder; and F, camera mounting pole. The camera trap is mounted ~70 cm above the tile with the PIR sensor oriented furthest from the mounting pole.

We deployed four Reconyx HC600 camera-traps to each 100 metre transect, positioning camera-trap stations at approximately the 20, 40, 60, and 80 m intervals (Fig 1). Each station comprised of: a camera-trap positioned ~70 cm above the ground (and associated mounting equipment) with the lens and passive infrared (PIR) sensor facing down; a cork floor tile (30 x 30 x 0.6 cm) positioned beneath the camera-trap with a PVC bait-holder; and two drift fences (30 x 300 cm) extending either side of the cork tile (Fig 2). Bait was used to lure mammals and does not affect squamate detections [17].

Reconyx HC600 camera-traps can be triggered by both PIR and time-lapse triggers [18]. As such, we set the PIR trigger to operate continuously on the highest sensitivity setting and captured three images for each trigger event, and set the time-lapse trigger to capture an image every 5 min between 0700 and 1900 h. Welbourne, Claridge [19] demonstrated that these settings provide a high level of effectiveness without excessive maintenance requirements. Given the relatively long deployment period, batteries were refreshed, and images downloaded when the labour-intensive methods sub-surveys were conducted. Detected fauna were identified to species level using reference images and texts [20].

### Labour-intensive methods protocol

The labour-intensive methods survey consisted of four sub-surveys between 23–26 November 2014, 9–12 December 2014, 6–9 January 2015, and, 1–4 February 2015. We conducted each sub-survey for four consecutive days following Fisher and Rochester [15] recommendations. In any case, only four consecutive days could be surveyed due to access limitations on BWR. At each transect, pitfall traps were established at approximately the 10, 30, 50, and 70 m intervals along the transect, and arrays of artificial refuges were positioned at approximately the 25 and 75 m mark (Fig 1). Pitfall traps were comprised of a white 20 L (Ø 28.5 x 31 cm) bucket buried with the brim level to the ground. Drift fences, identical to those used with the camera-traps (*i*.*e*., ~30 x 600 cm total with the bucket centrally located), were used at each pitfall trap. Vegetation, a small bait ball of peanut butter and oats, and a wet sponge were placed in the bottom of each pitfall trap to provide shelter and reduce trap mortality [15]. Each artificial refuge array consisted of four concrete roof tiles (2 x 25 x 40 cm), four wooden railway sleepers (12 x 25 x 125 cm), and two sheets of corrugated steel (~85 x ~120 cm) stacked one on top of the other (Fig 1).

Pitfall traps were installed in late October 2014 to allow the habitat to recover from trap installation [13]. Artificial refuges were established in January 2011 as part of a long-term study by Australian National University researchers. When not in use, pitfall traps were covered with the bucket lid and artificial refuges remained in place. Buckets were opened by 1600 h the day prior to the sub-survey beginning. Pitfall traps and artificial refuges were inspected twice per day (morning and afternoon), resulting in eight sampling occasions per sub-survey. Captured or observed animals were identified to species-level.

### Effectiveness

Given the three detection methods, and total survey length, we compared seven variants of survey protocol (Table 1). We determined the effectiveness of the survey protocols by calculating the probability that one survey protocol detected more species than another survey protocol. To do this, we first estimated the average number of species detected (*λ*_*ij*_) for the *i*^th^ survey protocol at the *j*^th^ transect using the following Bayesian hierarchical model:

$$s_{ij}\sim Pois(\lambda_{ij})$$

$$\text{ln}(\lambda_{ij})=\alpha +\beta_{i}+\gamma_{j}$$

**Table 1:** Survey protocol combinations.

Survey protocol	Description
All-74	Detections from all methods during the 74 days of surveying.
CT-74	Detections from 74 days of camera trapping.
All-16	Detections from all methods during the 16 days of labour-intensive methods survey.
CT-16	Detections with camera traps during the 16 days of labour-intensive methods survey.
LI	Detections from the artificial refuges and pitfall traps alone during the 16 days.
AR	Detections from artificial refuges alone.
PF	Detections from the pitfall traps alone.

Survey protocol combinations derived from camera trapping (CT) and labour-intensive (LI; *i*.*e*., artificial refuges AR and pitfall traps PF) detection methods.

We assumed the number of squamate species detected (***s***) was Poisson distributed, and *α* and *β* were sampled from vague normal distributions with *mean* = 0 and *variance* = 1E^12^. The transect was incorporated as a hierarchical (random) effect from the following distributions:

$$\gamma_{j}\sim N(0,{\theta_{j}}^{-2})$$

$$\theta_{j}\sim Unif(0,100)$$

The posterior distributions of the estimated number of species detect for each survey protocol were then used to calculate the probability that one survey protocol would detect more species than another, and the estimated number of species detected was used in cost-effectiveness calculations.

### Cost-effectiveness

Cost-effectiveness is a measure of performance that accounts for the financial cost of achieving said performance [21]. It is usually expressed as outcome per dollar input or alternatively the cost per unit of outcome. Here, we expressed cost-effectiveness as the average marginal cost per squamate species detected at the transect level since marginal cost will determine long-term total cost projections [22]. Survey costs were calculated independently for the camera-trapping survey and the labour-intensive methods survey from their associated equipment and implementation expenses (Table 2). Costs for the 16-day camera-trapping protocol (CT-16) were calculated assuming camera-traps were deployed and retrieved for each sub-sample, however camera-traps would ordinarily not be deployed in this manner.

**Table 2:** Equipment and implementation expenses to conduct a survey.

Labour-intensive methods	Camera traps
Equipment items	
Bucket with lid	Reconyx^™^ HC600
Drift fence	Memory card
Pegs	Drift fence
Sponge	Pegs
Roof tile	Floor tile
Railway sleeper	Mounting pole
Corrugated steel	Bait holder
Implementation items	
*Vehicle*	*Vehicle*
Deployment	Deployment
Transect inspection	Recovery
	Maintenance
*Consumables*	*Consumables*
Bait	Bait
	Batteries
*Labour*	*Labour*
Deployment (artificial refuges)	Deployment
Deployment (Pitfall traps)	Recovery
Transect inspection	Data recovery
Data entry	Image identification and processing

Equipment and implementation items required to conduct labour-intensive (artificial refuges and pitfall trapping) and camera trap surveys of terrestrial squamates.

Since survey effectiveness estimates were determined for the entire survey period (*i*.*e*., 23 November 2014 to 4 February 2015), the entire period was considered a single survey and costs calculated as such. Thus, the total cost (*T*_*i*_) of *m* such surveys with the *i*^*th*^ survey protocol, and the marginal cost (*M*_*i*_) of the *i*^*th*^ survey protocol were calculated from:

$$T_{i}(m)=E_{i}(1+r(m-1))+m(V_{i}+C_{i}+hL_{i})+hl_{i}+v_{i}$$

$$\frac{dT}{dM}=M_{i}={rE}_{i}+V_{i}+C_{i}+hL_{i}$$

where *E* = equipment cost; *V* = vehicle cost; *C* = cost of consumables; *L* = hours of labour required for the survey; *l* = hours of labour required to deploy artificial refuges and pitfall traps; *v* = vehicle cost to deploy artificial refuges and pitfall traps; *r* = rate of replacement (RoR) of equipment where 0 < *r* < 1; and, *h* = hourly rate in dollars.

Equipment costs included all expenses associated with acquiring and preparing equipment for deployment. Equipment costs were considered as a sunk cost, since they could not practically be recovered once purchases were made. Thus, RoR represented an estimated proportion of equipment that would need to be purchased in successive surveys to account for attrition, assuming equipment could be replaced at the equivalent price. Implementation costs represented expenses required to execute the survey. Implementation costs were calculated on a best-case scenario whereby fieldworkers did not need to remain in the field overnight to conduct surveys; thus, accommodation expenses were not included, and hours of labour represented the actual time researchers spent conducting survey tasks. Vehicle costs were calculated at a flat rate of $100 per vehicle day, which is less than commercial four-wheel-drive hire [23] but more than daily running costs of a privately-owned vehicle [24]. It was assumed that the cost of deploying replacement artificial refuge or pitfall trapping equipment would be minimal and labour for this was not included in the marginal cost calculation.

To ascertain the financial cost of each method, total and marginal costs were simulated by varying the equipment RoR and hourly labour rate. Rate of replacement was varied in 1% increments from 0%, which indicated no loss or damage to equipment, to 30%, which represented considerable equipment loss. Hourly wages were varied in $1 increments from $0, indicative of a survey team comprised entirely of volunteers, to $55 per hour, which was slightly less than the hourly wage of $55.55 received by a Level 5 Casual Research Assistant at the University of New South Wales [25]. All costs were represented in Australian dollars.

Analyses were conducted using *R* Version 3.4.2 [26]. The package *R2OpenBUGS* [27] was used in *R* to call *OpenBUGS* (Version 3.2.3) [28]. Bayesian models were run using two Markov Chain Monte Carlo (MCMC) chains. The *R* package *CODA* was used to assess chain convergence [29]. Convergence was confirmed by visually examining convergence and autocorrelation plots, and by using Gelman and Rubin’s convergence diagnostic test to ensure shrinkage of parameter estimates were < 1.05 [30]. Iterations for Bayesian models varied depending on convergence, and half of the iterations were used as burn-in. This study was conducted under the UNSW Animal Research Ethics Permits 12/14A and 14/141B.

## Results

Although the total camera-trap deployment was 74 days, five days were removed from the final camera-trap analysis due to maintenance. Additionally, although 40 camera-traps were deployed during the survey, three camera-traps, each at different transects, failed shortly after deployment and continued to fail after each maintenance event. Nevertheless, these transects were not removed from the analysis. Overall, camera-traps resulted in 2492 camera-trap days. The labour-intensive methods resulted in 960 trap days and 1920 trap inspections, consisting of 640 pitfall trap days and 320 artificial refuge ‘trap’ days. Across all protocols, 10 squamate species were detected, all of which were detected by camera-traps. In contrast, seven species were detected by artificial refuges and six squamate species were detected by pitfall traps (Table 3).

**Table 3:** Number of transects at which squamate species were detected.

Group and Scientific name	Common name	All-74	CT-74	All-16	CT-16	LI	AR	PF
Small lizards (SVL^a^ ≤ 50 mm)								
*Lampropholis delicata*	Delicate skink	10	10	10	10	10	10	7
*Saproscincus mustelinus*	Weasel skink	2	2	2	2	2	1	1
Medium–large lizards (SVL > 50 mm)								
*Acritoscincus platynota*	Red-throated skink	2	2	1	0	1	1	1
*Amphibolurus muricatus*	Jacky dragon	10	10	8	8	4	1	4
*Ctenotus taeniolatus*	Copper-tailed skink	8	8	4	4	2	0	2
*Cyclodomorphus michaeli*	Mainland she-oak skink	5	2	4	1	4	3	1
*Tiliqua scincoides*	Blue-tongue skink	6	6	2	2	0	0	0
Snakes								
*Hemiaspis signata*	Marsh snake	6	3	6	2	5	5	0
*Pseudechis porphyriacus*	Red-bellied black snake	7	7	3	3	0	0	0
*Pseudonaja textilis*	Eastern brown snake	4	3	3	2	2	2	0

Survey protocols are: All-74, detections from all protocols during the 74 days; CT-74, detections from camera traps during the 74 days; All-16, detections from all using all protocols during the 16 days of the labour-intensive methods survey; CT-16, detections from camera traps during the 16 days of the labour-intensive methods survey; and LI, combined detections of artificial refuges (AR) and pitfall traps (PF).

^a^Snout-vent length

### Detection effectiveness

**Fig 3:**
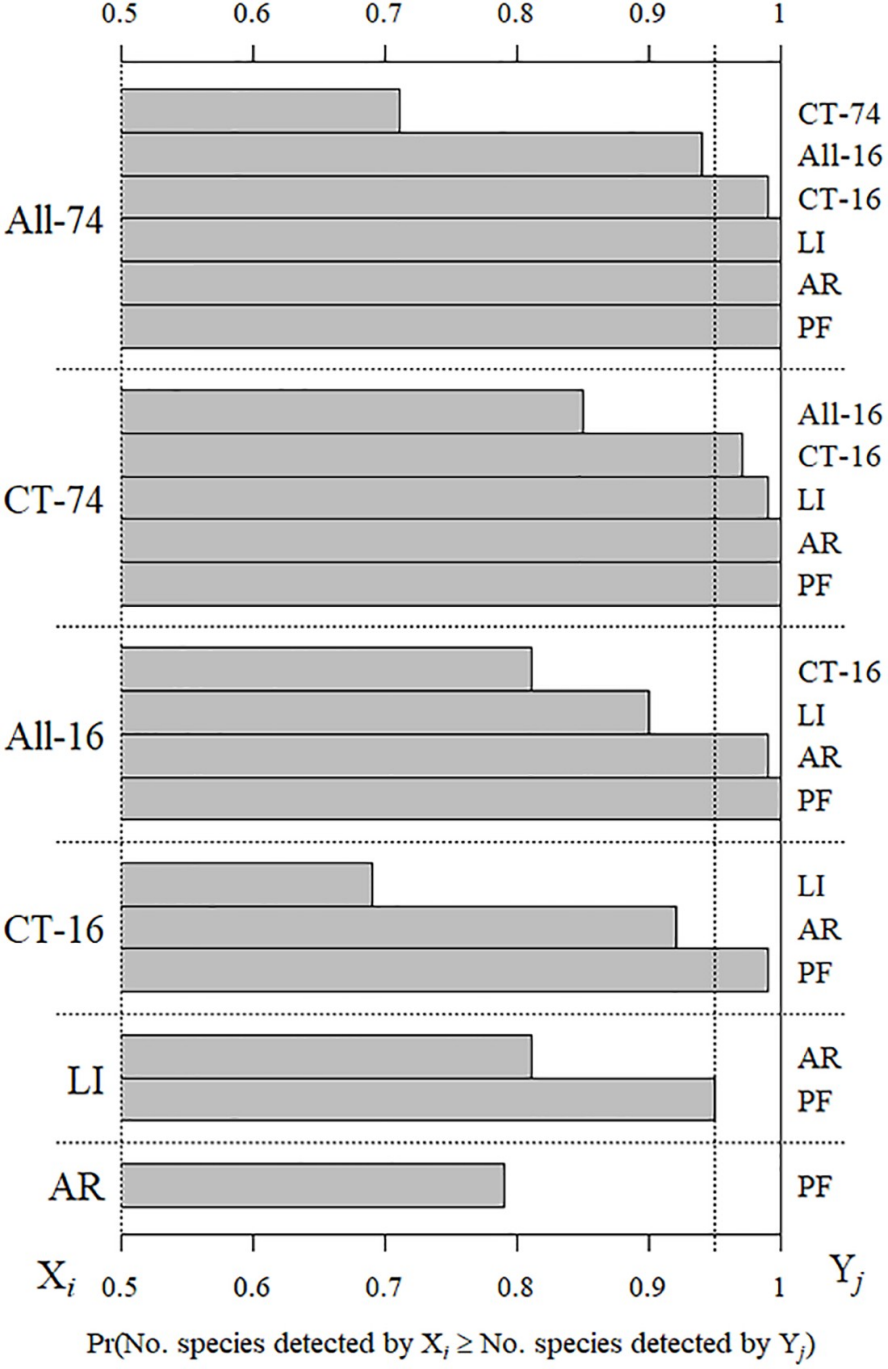
Comparison of survey protocols. Probability that the number of species detected by the *X*_*i*_ survey protocol was greater than or equal to the number of species detected by the *Y*_*j*_ survey protocol. Probabilities were calculated from 10000 draws from the survey protocol’s posterior distribution. Survey protocols are: All-74, detections from all protocols during the 74 days; CT-74, detections from camera traps during the 74 days; All-16, detections from all using all protocols during the 16 days of the labour-intensive methods survey; CT-16, detections from camera traps during the 16 days of the labour-intensive methods survey; and LI, combined detections of artificial refuges (AR) and pitfall traps (PF).

There was a 99% probability that the 74-day camera-trapping survey (CT-74) detected more squamate species per transect than artificial refuges and pitfall traps, either alone or together (*i*.*e*., labour-intensive methods, LI; Fig 3). There was a 71% probability that complementing the 74-day camera-trapping survey with 16-days of labour-intensive methods (*i*.*e*., All-74) would increase species detections per transect, yet the overall number of squamates detected was equivalent (Fig 3). The species influencing the transect level result were the mainland she-oak skink (*Cyclodomorphus michaeli*) and the marsh snake (*Hemiaspis signata*; Table 3). Although using all methods together for 16-days (*i*.*e*., All-16) detected the 10 squamate species, the 74-day camera-trapping survey detected more squamate species per transect ~85% of the time. Pitfall trapping alone was the least effective technique tested; however, detections with pitfall traps complemented detections with artificial refuges (Table 3).

### Cost-effectiveness

Initial equipment outlay was the most expensive component of the camera-trapping surveys, whereas implementation expenses were the most expensive component of the labour-intensive methods (Table 4). The equipment for labour-intensive methods cost only ~8.5% of the cost for camera-trapping equipment (Table 4). Depending on the cost of hourly labour, the first survey with labour-intensive methods cost between $4,700 and $17,845 ($0 and $55 per hour, respectively); while camera-traps cost between $27,482 and $29,737 for 74-days ($0 and $55per hour, respectively), and $27,498 and $30,325 for 16-days ($0 and $55 per hour). Using artificial refuges and pitfall traps simultaneously (*i*.*e*., labour-intensive methods) was less costly than using both protocols individually, as vehicle and labour costs were not duplicated. This was not the case for including camera-trapping with the labour-intensive methods (*i*.*e*., All-16, All-76), as most costs were additive.

**Table 4:** Equipment and implementation expenses.

Item	All-74	CT-74	All-16	CT-16	LI	AR	PF
Equipment ($)	28862	26582	28862	26582	2280	1160	1120
Vehicle ($)	2000	400	2000	800	2000	2000	2000
Consumables ($)	620	500	236	116	120	0	120
Labour (h)	225	41	235	51	184	173	176
Labour^a^ (h)	55		55		55	15	40
Vehicle^a^ ($)	300		300		300	300	300

Equipment and implementation expenses (rounded to the nearest dollar) for the various survey protocols for terrestrial squamates. Survey protocols are: All-74, detections from all protocols during the 74 days; CT-74, detections from camera traps during the 74 days; All-16, detections from all using all protocols during the 16 days of the labour-intensive methods survey; CT-16, detections from camera traps during the 16 days of the labour-intensive methods survey; and LI, combined detections of artificial refuges (AR) and pitfall traps (PF).

^a^Initial deployment

**Fig 4:**
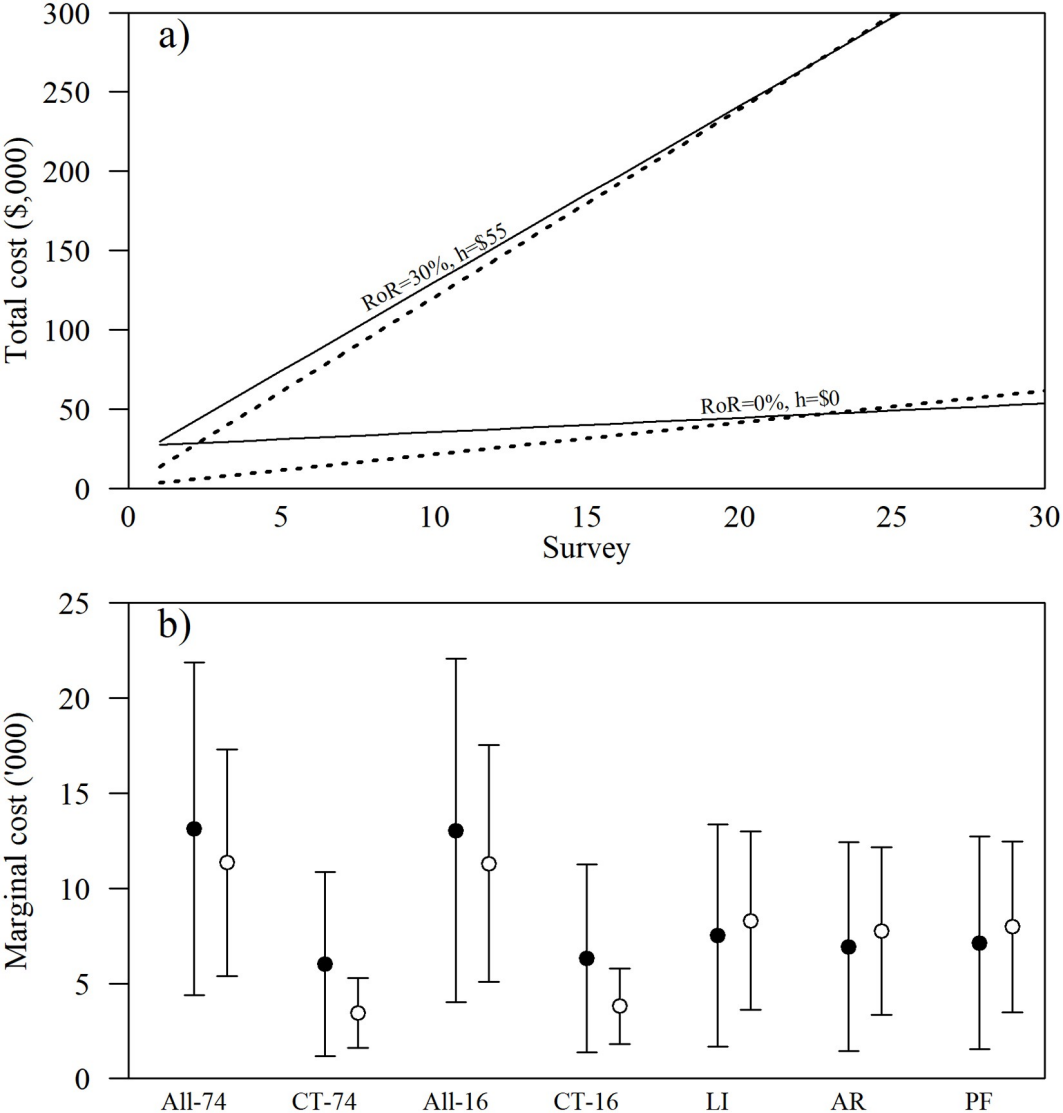
Total cost and marginal cost for terrestrial squamate surveys with various survey protocols. Plot a) depicts the total cost for the 74-day camera trapping protocol (CT-74; solid line) and artificial refuges alone (AR; dashed line) at two rate-of-replacement (RoR; 0 and 30%) and hourly rate of labour (h; $0 and $55) values. Plot b) depicts the average marginal cost over all values of RoR and hourly labour (black dots) and average marginal cost for a realistic scenario whereby RoR is < 10% and hourly wage > $10 (white dots). Survey protocols are: All-74, detections from all protocols during the 74 days; CT-74, detections from camera traps during the 74 days; All-16, detections from all using all protocols during the 16 days of the labour-intensive methods survey; CT-16, detections from camera traps during the 16 days of the labour-intensive methods survey; and LI, combined detections of artificial refuges (AR) and pitfall traps (PF). Dots in plot b) represent means and bars 95% confidence interval.

The total cost of successive surveys after the first survey varied greatly depending on the RoR and cost of hourly labour (Fig 4). Over all scenarios of RoR and hourly labour, the mean marginal cost of using camera-traps for a 74-day survey (mean *M*_*CT-74*_ = 6014, *SD* = 2469) was less than the means of all other survey protocols (Fig 4b). Consequently, all else being equal, the 74-day camera-trapping survey protocol was overall less costly than other methods in the long-term. For example, Fig 4a shows the total cost of the 74-day camera-trapping protocol and artificial refuges alone at two RoR and hourly labour values, and in both cases camera-traps are less costly in the long-term. Camera-trapping was more costly than other methods where RoR was very high (*i*.*e*., > 20%) and hourly labour was very low (*i*.*e*., < $5), or where RoR of the camera-trapping protocol was much higher than the RoR of the labour-intensive methods protocol. Still, under a realistic scenario where RoR is < 10% and cost of hourly labour is > $10 camera-trapping is less costly than labour-intensive methods (Fig 4b).

**Fig 5:**
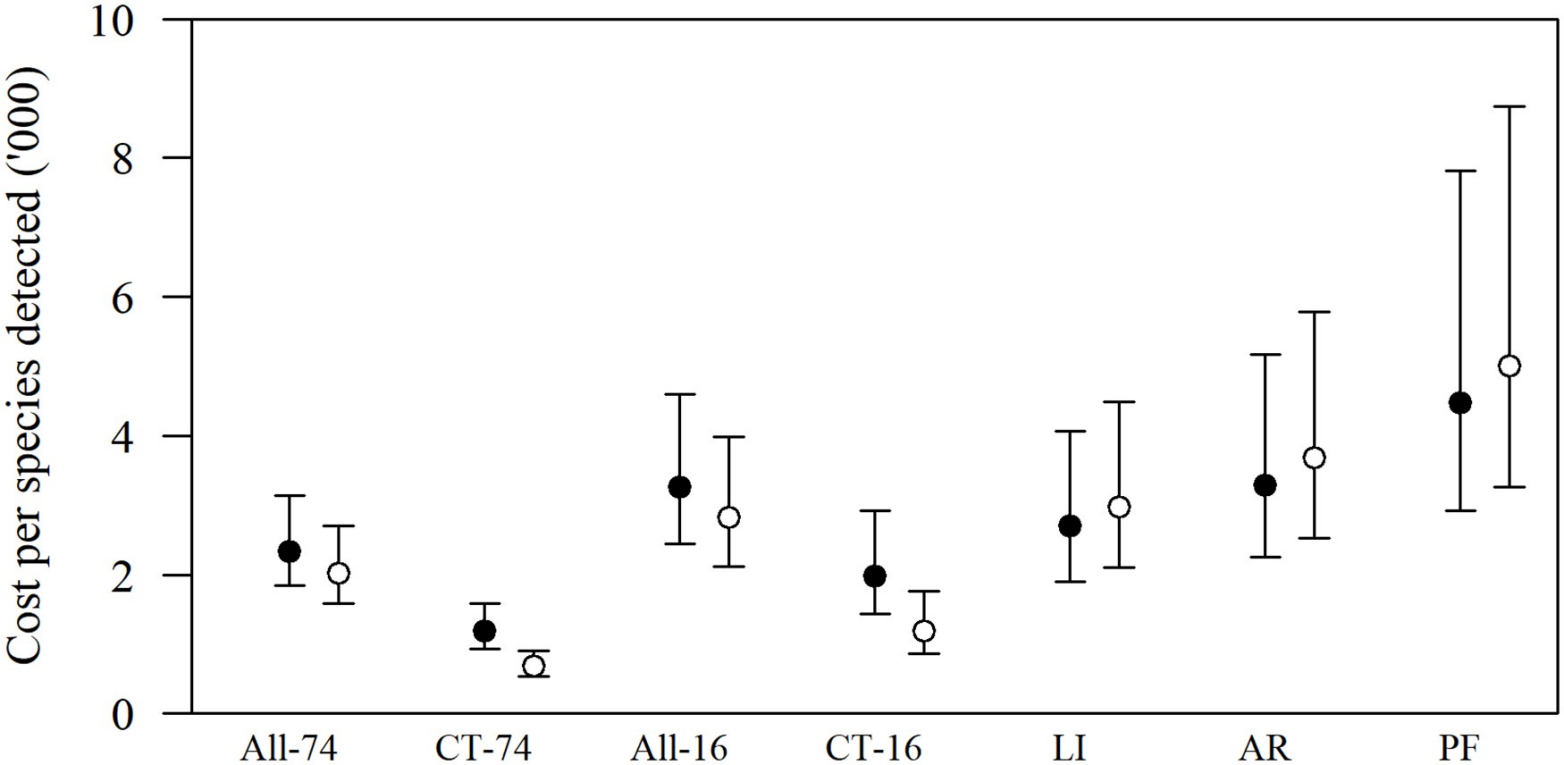
Cost ($’000) per squamate species detected as a function of survey protocol. Black dot estimates are calculated from the average marginal cost over all values of RoR and hourly labour, and white dots are calculated from the average marginal cost for a realistic scenario whereby RoR is < 10% and hourly wage > $10. Survey protocols are: All-74, detections from all protocols during the 74 days; CT-74, detections from camera traps during the 74 days; All-16, detections from all using all protocols during the 16 days of the labour-intensive methods survey; CT-16, detections from camera traps during the 16 days of the labour-intensive methods survey; and LI, combined detections of artificial refuges (AR) and pitfall traps (PF). Central dots represent the mean of the posterior distribution and bars represent 95% credible intervals.

By combining the average marginal cost with species detection estimates, the 74-day camera-trapping protocol was most cost-effective (Fig 5). Based on all RoR and hourly labour values, the 74-day camera-trapping protocol cost $1198 (CI 931–1590) per species detected, whereas the labour-intensive methods protocol cost $2698 (CI 1907–4068) per species detected. The difference in cost-effectiveness between these survey protocols widened under a realistic scenario of RoR < 10% and cost of hourly labour > $10 (Fig 5, white dots). Under a realistic scenario, the 74-day camera-trapping protocol cost $687 (CI 534–912) whereas the labour-intensive methods cost $2975 (CI 2103–4486) per species detected at the transect level. Although supplementing camera-traps with labour-intensive methods can improve detections at the transect level (Fig 3), using all methods together (*i*.*e*., All-16 and All-74) is less cost-effective than camera-trapping alone (Fig 5).

## Discussion

To better understand ecological systems and how best to manage them, it is vital that cost-effective methods for detecting wildlife are developed. Squamates are a highly diverse vertebrate group that are data poor, due in part to their cryptic nature and subsequent difficulty in detecting them. Camera-traps may be a useful tool for detecting squamates and here we compared the effectiveness and cost of using camera-traps to survey a terrestrial squamate assemblage to two commonly employed methods: artificial refuges and pitfall traps. Our findings indicate that camera-traps are both more effective and more cost-effective than the labour-intensive methods evaluated for surveying a squamate assemblage; and, further refinements of camera-trapping methods are required for detecting nocturnal squamates. These findings have several implications for wildlife researchers and managers. First, those wildlife researchers and manager currently using camera-traps to survey non-squamate fauna, can incorporate squamate surveys into current projects. Secondly, squamate researchers using existing methods can confidently transition to or incorporate camera-traps as a survey device. Third, camera-traps are not a panacea, as no method is, and practitioners will need to evaluate their circumstances and the study environment before adopting a camera-traps.

The most important finding is that the 74-day camera-trapping protocol (CT-74) is effective and more cost-effective than the labour-intensive methods tested for inventorying a squamate assemblage. While using camera-traps and labour-intensive methods in combination may increase the number of species detected at the transect level (All-74), it may not improve overall detections and has considerable financial and ethical (see below) costs associated. Consequently, those wildlife researchers and managers currently using camera-traps to survey non-squamate fauna can incorporate squamate surveys into their fauna monitoring with few additional equipment requirements (*i*.*e*., drift fence, cork tile). For those researchers using traditional methods to survey squamates, this study supports a growing body of literature demonstrating that camera-traps present a new tool for answering existing questions and asking new questions [8–10, 31]. Additionally, other camera-trap designs may further improve the effectiveness of this approach.

Although camera-traps are more effective, they are initially more expensive and require a number of surveys to be carried out before becoming less costly overall. The specific number of surveys will depend upon the research context; for example, if field researchers are volunteers or paid technicians. Additionally, in some situations camera-traps can be more expensive than labour-intensive methods. Due to the high equipment cost of camera-traps, high rates of theft can result in overall higher costs compared with other methods. We did not experience theft of any equipment, but other researchers have reported numerous camera-traps stolen or damaged during surveys [32]. Attempts have been made to reduce theft through signposting [33], installing security posts [34, 35], and changing deployment strategies [32], with varying levels of success and applicability. Wildlife researchers and managers will need to evaluate their deployment circumstances and associated costs carefully before solely adopting camera-traps.

That said, these results understate the financial advantage of using camera-traps, in several ways. First, we calculated survey costs assuming field researchers would not be required to stay in the field. This assumption greatly reduced the cost of the labour-intensive protocols relative to camera-traps. Labour-intensive methods typically require field researchers to remain in the field for protracted periods, often with associated financial costs for incidentals and accommodation, whereas camera-traps can be deployed, maintained, and retrieved in shorter timeframes. Second, detections and costs associated with camera-trapping surveys are less susceptible to adverse weather in the following sense. During one of the labour-intensive methods sub-surveys, heavy rain prohibited pitfall traps from being opened. Surveying during that occasion was abandoned and conducted the following week. The two days that field staff spent at the site were not included in cost calculations in this study since we aimed to compare camera-traps to the best possible case of using traditional methods. Yet, ordinarily these costs would be part of the overall survey liability. Additionally, detections of squamates can be impacted by weather [36]. Read and Moseby [37] advised that eight sample days are required when surveying squamates with pitfall traps to ameliorate weather effects. As camera-traps are deployed for longer periods, inventory results are generally less biased since short-term weather effects become less important to the survey.

While camera-traps were more effective overall, the current methodology is limited. The labour-intensive methods detected *C*. *michaeli* and *H*. *signatai* at more transects than did camera-traps. This is most likely related to the behavioural ecology of these two species. Although *C*. *michaeli* will bask during the day, it is normally crepuscular [38]. Similarly, *H*. *signata* is active both day and night, but it is often nocturnal during warm periods [39, 40], such as in summer when this survey was conducted. The poor detection of these species with camera-traps reinforces findings by Richardson, Nimmo [9], who found that camera-traps were not effective for detecting nocturnal species. Given the way PIR triggered camera-traps operate [41] this is unsurprising, but clearly needs addressing to further broaden the utility of the method. Perhaps the simplest solution to this limitation is to program camera-traps to capture time-lapsed images throughout the night.

Further improving effectiveness with the methods tested can be achieved in two ways: modifying the detection device; and/or, increasing survey effort. While the equipment setup in the current experiment was apt for the comparison, the setup of both camera-traps and pitfall traps was likely less than optimal than if these methods were used in isolation. Drift fence lengths and array designs have been the focus of considerable research [15]. As camera-trapping and pitfall trapping are similar, that is each method requires an organism to enter a specific area to generate detections/captures, then many rules relating to optimal drift fence use with pitfall traps are likely applicable to camera-trapping. Hence, camera-traps on linear transects should probably be best established as continuous trap-lines rather than separate stations [42]. Furthermore, where sampling is not confined to transects, using cross-shaped rather than linear arrangements of drift fence may prove more effective [43].

Pitfall traps of the size used here are more effective at capturing small lizards than large lizards or snakes [44]. Therefore, observing that pitfall traps did not capture snakes was unsurprising. But for *L*. *delicata*, which is apparently abundant and among the smallest squamate at this location [20], pitfall traps performed poorly compared to camera-traps. This is intriguing since the arrangement of pitfall traps and camera-traps were functionally identical. Both devices direct fauna to a central area where they are captured or detected. The ground area photographed with a camera-trap was ~2000 cm^2^, and the tile occupies ~900 cm^2^ where most detections occur. The pitfall trap opening is ~71% smaller than the tile, covering ~638 cm^2^. Thus, a component of the difference in detection of *L*. *delicata* between these methods was likely due to the spatial extent being sampled, but given the stark disparity, much of the difference may result from trap-avoidance, which has been observed in studies of other species (*e*.*g*., Komodo monitors, *Varanus komodoensis*, [6]). Alternatively, perhaps the cork tile in the camera-trapping setup is acting as a heat lure. If either or both effects are at play, detections with these methods may be biased.

Increasing survey effort can be achieved by increasing the number of sampling stations at a sampling location and/or by increasing the number of days of sampling. In this respect camera-traps have a considerable advantage over labour-intensive methods if the number of days of sampling are to be extended. Once deployed, there is little extra cost associated with leaving camera-traps in the field for longer periods. Still, it is important to note that extending the number of sampling days cannot be done indefinitely as assumptions of population closure will become an issue and invalidate certain modelling approaches [45, 46]. Increasing the number of sampling stations at each sampling location is clearly possible and future research should examine this aspect more carefully. Here, we found that three camera-traps per transect resulted in the same number of squamate species detected as four camera-traps [17]. Again, prior research on pitfall trapping arrangements and density may be instructional.

One final point goes to the issue of animal welfare. The camera-trapping approach presented here appears less impactful than pitfall trapping. Mortality of fauna in pitfall traps, due to exposure or predation, is widely discussed in the literature [47–52]. Our study reinforces those concerns. Despite checking pitfall traps twice daily and despite including vegetation, wet sponges, and food in pitfall traps, several small lizards and eight frogs perished during the survey as a direct result of being captured. Of course, camera-traps, and for that matter artificial refuges, should not necessarily be considered as being impact free. Most squamate survey methods, including visual only methods, can result in some form of negative impact [53, 54]. Camera-traps and artificial refuges, which require the fauna to be concentrated into particular area, may increase predation that would be ordinarily avoided. It is unlikely that camera-traps impact target and non-target species in the manner that trapping methods demonstrably do, but wildlife researchers and managers should still consider potential impacts, especially where dealing with sensitive or threatened species.

## Conclusion

We compared the cost-effectiveness of using camera-traps with methods commonly used to survey a squamate assemblage and found camera-traps were more effective and clearly more cost-effective. These results demonstrate that, under the given circumstances, camera-traps provide wildlife researchers and managers with a versatile tool with which to inventory squamates. Still, camera-traps are not a panacea. Researchers need to closely assess their requirements before investing in camera-trapping equipment or adopting camera-traps for surveying squamates. Important considerations are the species assemblage, the environment in which sampling is to be conducted, and the resource at the researcher’s disposal. Continued research is required to overcome the identified limitations of surveying squamates with camera-traps, particularly issues relating to detection of crepuscular and mostly nocturnal species. By further developing camera-trapping methods, and perhaps adopting lessons learnt from pitfall trapping, camera-traps may help fill some of the fundamental missing data on squamates.
